# Status and outlook of mRNA therapeutics for viral diseases

**DOI:** 10.1038/s44321-026-00390-z

**Published:** 2026-02-11

**Authors:** Qiuwei Pan, Wenshi Wang, Harry L A Janssen, Zifu Zhong

**Affiliations:** 1 Department of Gastroenterology and Hepatology, Erasmus MC-University Medical Center, Rotterdam, The Netherlands; 2 Department of Pathogen Biology and Immunology, Jiangsu Key Laboratory of Immunity and Metabolism, Jiangsu International Laboratory of Immunity and Metabolism, Xuzhou Medical University, 221004 Xuzhou, China; 3 Toronto Center for Liver Disease, Toronto General Hospital, University of Toronto, Toronto, ON Canada; 4 Department of Pharmaceutics, Ghent University, Gent, 9000 Belgium; 5 Present Address: David H. Koch Institute for Integrative Cancer Research, Massachusetts Institute of Technology, Cambridge, MA USA

**Keywords:** mRNA Therapeutics, Drug Delivery, Viral Diseases, Immunology, Microbiology, Virology & Host Pathogen Interaction, RNA Biology

## Abstract

Endemic and emerging viral diseases continue to impose significant health, economic, and societal burdens worldwide. Vaccines and therapeutics represent two key pillars in the fight against these threats. Since the clinical success of mRNA vaccines during the COVID-19 pandemic, mRNA therapeutics have rapidly evolved from a niche innovation into a validated and versatile medical platform. While early efforts focused primarily on vaccine development, recent advances have expanded the scope to antiviral applications of in vitro-transcribed mRNA. Emerging strategies include in vivo expression of neutralizing antibodies for passive immunization, delivery of innate immune effectors such as interferons and antiviral peptides, and programmable CRISPR-based antiviral systems. In parallel, progress in mRNA delivery technologies has enabled clinical translation, although challenges related to stability, specificity, and immunogenicity remain. In this Perspective article, we review recent preclinical and clinical advances in mRNA therapeutics for viral infections. We also highlight key scientific, technical, and regulatory challenges, and propose strategic solutions to address the pressing need for controlling endemic viral diseases and enhancing global pandemic preparedness.

## Abstract (web summary)

In this perspective, Q. Pan, Z. Zhong and colleagues discuss recent advances in mRNA therapeutics for antiviral applications, highlighting emerging strategies, delivery improvements, and key challenges in controlling viral diseases and improving pandemic preparedness.

## Graphical abstract

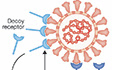

## Principles of mRNA technology and mRNA therapeutics

**Figure 1:**
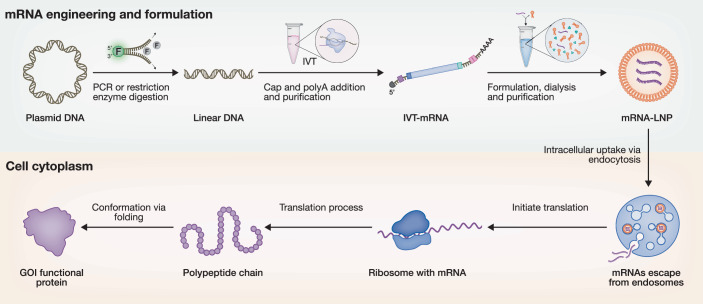
Schematic representation of in vitro manufacturing and translation of therapeutic mRNA. The engineering and manufacturing process of synthetic in vitro-transcribed (IVT) mRNA, its formulation into lipid nanoparticles (mRNA–LNPs), and the subsequent intracellular delivery pathway. Upon cellular uptake, the mRNA is translated into functional proteins corresponding to the gene of interest (GOI). The figure was created using BioRender.

Principles of messenger RNA (mRNA) technology center on designing synthetic mRNA that can be efficiently delivered into target cells to produce desired proteins. In the early 1970s, mRNA-based gene transfer was pioneered by investigating its uptake and effects in mammalian cells (Bhargava and Shanmugam, 1971). In 1989, a landmark study first demonstrated its therapeutic potential, confirming that liposome-encapsulated mRNA could drive protein expression in cells (Malone et al, 1989). This breakthrough was soon followed in the early 1990s by in vivo validation, where direct administration of mRNA into animal models demonstrated efficient protein production (Jirikowski et al, 1992; Wolff et al, 1990). These pioneering findings established that in vitro-transcribed (IVT)-mRNA could be explored as an attractive therapeutic strategy that holds many advantages. Unlike plasmid DNA, mRNA avoids the risk of genomic integration, highlighting its promise as a safe and novel drug class (Pardi et al, 2018; Zhong et al, 2018) (Fig. 1).

Historically, mRNA was considered unstable for therapeutic application because it can be rapidly degraded by RNases. In recent years, however, advances in mRNA modification, structural optimization, and delivery systems have greatly endowed the molecule with enhanced structural and translational stability. Lipid nanoparticles (LNPs) are developed as a safe, efficient, and clinically validated non-viral delivery system that encapsulates nucleic acids by electrostatic association with ionizable lipids and releases their cargo within endosomes at acidic pH (Simonsen, 2024) (Fig. 1). In 2018, Patisiran became the first approved LNP-based siRNA drug for the treatment of hereditary transthyretin-mediated amyloid polyneuropathy (Akinc et al, 2019). Since then, IVT-mRNA platforms have rapidly advanced, exemplified by the two approved mRNA–LNP vaccines in responding to the COVID-19 pandemic (Schoenmaker et al, 2021). The field has since expanded, with additional approvals including ARCT-154, a self-amplifying RNA (saRNA) vaccine against SARS-CoV-2 in 2023 (Wayne and Blakney, 2024), and Moderna’s respiratory syncytial virus (RSV) vaccine, mRESVIA, approved in 2024. Beyond conventional mRNA, next-generation RNA platforms, including saRNA (Zhong et al, 2019), trans-amplifying RNA (taRNA) (Perkovic et al, 2023), and circular RNAs (circRNAs) (Chen et al, 2023), have emerged to broaden applications for protein expression (Box 1).

By enabling direct protein synthesis within the body and eliminating the need for large-scale protein manufacturing, therapeutic mRNAs are attracting significant scientific and clinical attention, though several major challenges remain. Moving beyond mRNA vaccines, mRNA-based therapeutic approaches are now being explored for a broad spectrum of diseases, in particular viral infections.

Box 1 Evolution of in vitro-transcribed (IVT)-mRNA platforms IVT-mRNA technologies have evolved from linear, short-lived transcripts to more durable and efficient designs.

- Conventional mRNA: Linear, capped, and polyadenylated transcripts optimized with modified nucleotides and untranslated regions (UTRs); clinically validated in vaccines and protein replacement therapies.
- Self-amplifying RNA (saRNA): Incorporates viral replicase for intracellular RNA amplification, enabling high protein expression at reduced doses.
- Trans-amplifying RNA (taRNA): Separates replicase and target protein-encoding RNAs into distinct molecules, improving modularity, safety, and manufacturing flexibility.
- Circular RNA (circRNA): Covalently closed structure resistant to exonuclease degradation; supports prolonged and efficient protein expression via internal ribosome entry sites (IRES) or rolling-circle elements.

## Opportunities and strategies of mRNA therapeutics for combating viral diseases

Endemic and epidemic viral diseases continue to impose significant public health, economic, and societal burdens worldwide. Although vaccines are widely recognized as the primary preventive measure against viral infections, effective therapeutics constitute an equally crucial component of the response to viral diseases. However, therapeutic development by major pharmaceutical companies is prioritized for only a small fraction of the more than 200 virus species responsible for significant clinical burdens, primarily in high-income countries (Chung and Baumert, 2014). Consequently, the majority of viral diseases remain neglected and lack viable treatment options. Recent advances in mRNA technology have opened unique opportunities for the development of both vaccines and therapeutics.

**Figure 2:**
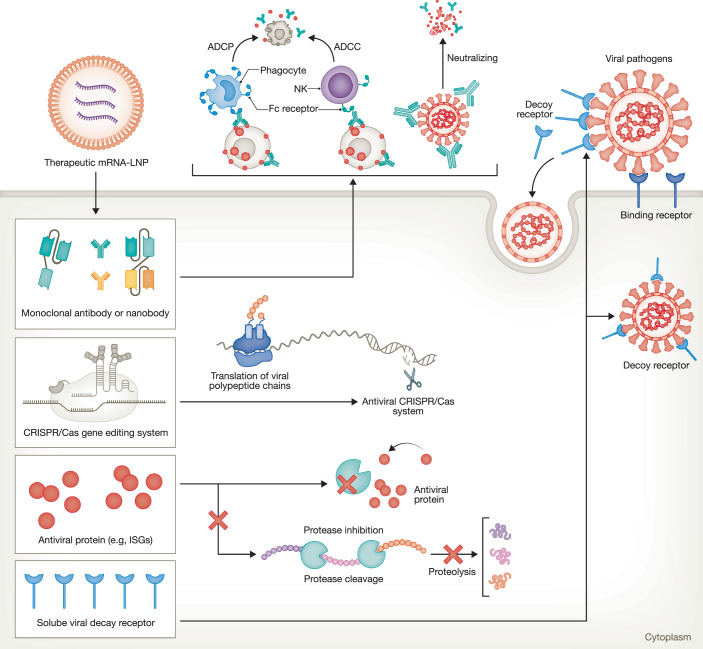
Schematic overview of therapeutic mRNA strategies for combating viral infections. The illustration highlights key approaches, including in vivo expression of monoclonal antibodies for passive immunization, delivery of host antiviral proteins, programmable CRISPR-based therapies, and soluble viral decoy receptors. These modalities exert antiviral effects through distinct mechanisms of action, including preventing viral entry, inhibiting viral replication, and cleaving viral genetic materials. ADCP antibody-dependent cellular phagocytosis, ADCC antibody-dependent cellular cytotoxicity, ISGs interferon-stimulated genes. The figure was created using BioRender.

Unlike mRNA vaccines, which are primarily designed to elicit long-term adaptive immune responses, therapeutic mRNAs are typically engineered as flexible antiviral interventions to enable rapid and transient production of key regulatory proteins that are directly involved in antiviral defense. These approaches hold particular promise for providing immediate, programmable, and highly targeted protection, especially in individuals unable to mount effective vaccine responses or who remain unvaccinated. Hereby, we highlight three major therapeutic strategies: expression of monoclonal antibodies (mAbs) for in vivo passive immunization, delivery of host antiviral effectors and cytokines, and programmable CRISPR-based antivirals (Fig. 2; Table 1).

**Table 1:** Overview of representative mRNA therapeutics for viral diseases in preclinical development.

mRNA therapeutics	Viral pathogen	Delivery approach	Animal model	Administered dose and route	Key notes	References
VRC01 mRNA encoding mAb	HIV	LNP	BALb/c mice and C57BL/6c mice	IV 0.3–1.4 mg/kg	• Single systemic dose (1.4 mg/kg mRNA) produces ~170 µg/mL VRC01 antibody in mouse plasma within 24 h. • Weekly dosing (1 mg/kg mRNA) in immunodeficient mice maintains VRC01 trough levels >40 µg/mL. • A single VRC01 mRNA injection protects humanized mice from intravenous HIV-1 challenge.	Pardi et al, 2017
Palivizumab encoding mRNA	RSV	Viromer RED; jetPEI;	BALB/c mice	IM, IT, 15 mg/kg	• Palivizumab mRNA expression reduces RSV F copies by 90.8%. • Engineered membrane-anchored palivizumab blocks detectable infection in transfected cells and reduces in vitro titer by 99.7% and in vivo RSV F copies by 89.6%. • Expressed anchored (aVHH) and secreted (sVHH) high-affinity camelid anti-RSV F antibodies	Tiwari et al, 2018
mRNA encoding 8-9D	SARS-CoV-2	LNP	C57BL/6c mice	IV 5 μg	• Identified 8-9D, a broadly neutralizing antibody against multiple SARS-CoV-2 variants, with its mechanism defined by structural analysis. • Lung-selective mRNA delivery enables high local expression of 8-9D and effectively blocks viral entry. • This approach protects mice from infections by Beta and Omicron variants, demonstrating strong potential for lung-targeted mRNA antibody prophylaxis.	Tai et al, 2023
mRNA encoding XGv264	SARS-CoV-2	LNP	mice and non-human primates	IV, 0.6 mg/kg, 0.3 mg/kg	• XGv264 mRNA-LNP is well-tolerated in non-human primates, achieving 3.3–9.6 μg/mL IgG after a single 0.3–1 mg/kg dose with strong, sustained neutralizing activity against SARS-CoV-2. • In macaques, a single prophylactic dose (0.3 or 0.6 mg/kg) provided dose-dependent protection against Omicron infection in both the upper and lower respiratory tracts	Chi et al, 2025
mRNA encoding mAbs	Orthopoxviruses, vaccinia virus,	LNP	BALB/c mice	IV, 1 mg/kg	• four mRNA–LNP constructs encoding broadly neutralizing anti-orthopoxvirus antibodies for rapid in vivo antibody expression after a single IV dose in mice. • mRNA-delivered antibodies protected mice from weight loss and mortality in a lethal vaccinia virus challenge. • A dual-targeting mRNA antibody cocktail (Mix2a) showed the strongest protection by simultaneously neutralizing viruses	Chi et al, 2024
mRNA encoding BD55-1205 mAb	SARS-CoV-2	LNP	Tg32 mice	IV, 0.5 mg/kg	• mRNA–LNP delivery of BD55-1205 in mice generated strong neutralizing responses (NT50 ≈ 5,000) against XBB.1.5, HK.3.1, and JN.1	Jian et al, 2025
mRNA encoding human IFNλ1 (ETH47)	SARS-CoV-2	LNP	K18-hACE c57BL/6 J mice	IN, 0.38 mg/kg	• ETH47 induces dose-dependent target protein expression and activates interferon-stimulated genes. • Pre-treatment with ETH47 lowers SARS-CoV-2 viral load both in vitro and in vivo. • ETH47 administration enhances survival in mice after lethal SARS-CoV-2 challenge.	Macht et al, 2024
mRNA encoding ISGs	Zika virus, influenza virus, SARS-CoV-2	LNP	C57BL/6 mice; Syrian golden hamsters	IN, IT; Dose: not available	• A 10-ISG mRNA collection was identified that recapitulates the broad antiviral potential of IFN-I, increasing resistance to Zika virus, VSV, and SARS-CoV-2 in IFN-I–nonresponsive cells. • LNP-encapsulated 10-ISG mRNA provides prophylactic protection in animal models, reducing influenza A plaque size and protecting hamsters from lethal SARS-CoV-2, highlighting its potential as a broad-spectrum antiviral strategy.	Akalu et al, 2025
CRISPR-Cas9 mRNA	HBV	LLNs	C57BL/6 mice	IV, Cas9 (0.56 mg/kg) and sg21 (0.25 mg/kg)	• LLNs delivered Cas9 mRNA + sgRNA to the liver and suppressed HBV markers (reduced HBsAg/HBeAg and liver HBV RNA/DNA) and produced detectable indels at target sites in vivo.	Jiang et al, 2017
CRISPR-Cas13 mRNA	influenza virus, SARS-CoV-2	PBAE-based polymer	BALB/c mice; Golden Syrian Hamsters	nebulizer delivery, 125 μg Cas13a mRNA and gRNA	• Delivered polymer-formulated Cas13a mRNA with validated guides to the respiratory tract via nebulization, enabling efficient pulmonary delivery. • Cas13a treatment reduces influenza virus and SARS-CoV-2 replication in mice and hamsters, respectively, indicating strong potential for mitigating respiratory viral infections.	Blanchard et al, 2021
CRISPR-Cas9 mRNA	HBV	LNP	C57BL/c mouse and tree shrew models	IV, 3 mg/kg Cas9 mRNA and gRNA	• Cas9 mRNA + gRNAs mRNA-LNPeffectively edited HBV DNA in vivo, reducing HBcAg, HBsAg, and cccDNA by 53–73% in AAV-HBV mice. • In HBV-infected tree shrews, treatment lowered viral RNA by 70% and cccDNA by 35%; in HBV-transgenic mice, it suppressed HBV RNA by 90% and HBV DNA by 95%. • The CRISPR-LNP therapy was well-tolerated across species, showing no liver toxicity and minimal off-target effects in vivo.	Yi et al, 2023

*LNP* lipid nanoparticle, *LLP* lipid-like nanoparticle, *IV* intravenous, *IN* intranasal, *IM* intramuscular, *IT* intratracheal, *IFN* interferon, *ISG* interferon-stimulated gene.

### Expression of monoclonal antibodies for in vivo passive immunization

mAbs combat viral infections either by directly neutralizing virions or by engaging Fc-mediated effector functions such as antibody-dependent cellular cytotoxicity (ADCC), phagocytosis, and complement activation (Pantaleo et al, 2022). Despite their therapeutic potential, clinical application of mAbs is often constrained by their fragility, high production costs, and logistical challenges in distribution. In contrast, mRNA-encoded mAbs present a promising alternative, enabling host cells to transiently produce functional antibodies in situ. This approach offers rapid protection and adaptability against emerging viral threats. Notably, mRNA expression typically persists for days to weeks, depending on the platform, and the resulting antibodies may remain active longer than those delivered as recombinant proteins (Kowalski et al, 2019; Zhong et al, 2018).

To minimize innate immune activation, mRNA sequences are commonly modified with nucleosides such as 1-methylpseudouridine (m1Ψ) (Schoenmaker et al, 2021). For instance, m1Ψ-modified mRNAs encoding the heavy and light chains of the broadly neutralizing anti-HIV-1 antibody VRC01 have been formulated in LNPs (Pardi et al, 2017). In murine models, systemic administration of VRC01 mRNA–LNPs led to sustained plasma antibody levels, and a single injection protected humanized mice from intravenous HIV-1 challenge (Pardi et al, 2017). Building on this success, preclinical studies have expanded the approach to other viral pathogens, including RSV (Tiwari et al, 2018), SARS-CoV-2 (Tai et al, 2023), orthopoxviruses (Chi et al, 2024), and influenza virus (Vu et al, 2025). Interestingly, mRNA-encoded neutralizing mAbs such as XGv264 and BD55-1205, which were originally isolated from COVID-19 patients or COVID-19 vaccine recipients, conferred effective protection against SARS-CoV-2 in rhesus monkeys (Chi et al, 2025; Jian et al, 2025). A first clinical proof-of-concept emerged in 2019 with Moderna’s mRNA-1944, an LNP-formulated mRNA encoding a human neutralizing antibody targeting chikungunya virus (August, Attarwala et al, 2021). In a phase 1 trial, adverse events were reported to be mild to moderate and non-serious. Intravenous administration of mRNA-1944 (0.1–0.6 mg kg^−1^) resulted in durable antibody expression in vivo and robust neutralization ex vivo, further supporting its therapeutic potential. Administration of 0.3 or 0.6 mg kg^−1^ mRNA-1944 persisted for over 16 weeks with neutralizing activity (August et al, 2021). Currently, several pharmaceutical companies, including Moderna and BioNTech, are actively advancing pipelines of mRNA-encoded monoclonal or bispecific antibodies in clinical development (August et al, 2021; Stadler et al, 2024) (Table 2).

**Table 2:** Overview of mRNA therapeutics in clinical development.

mRNA therapeutics	Developer	Indication	Approach	Phase	Key notes	Clinical trial identifier	References
mRNA-1944	Moderna	Chikungunya virus (CHIKV) infection	Monoclonal antibody	Phase I	• An mRNA–LNP formulation encoding the heavy and light chains of a CHIKV-specific monoclonal neutralizing antibody. • A Phase 1 clinical study demonstrated systemic expression of functional antibodies with ex vivo neutralizing activity and an acceptable safety profile.	NCT03829384	August et al, 2021
BNT142	BioNTech	Cancer	Bispecific antibody	Phase I/IIa	• An mRNA–LNP therapeutic encoding a bispecific antibody targeting CLDN6 and CD3 is being developed for patients with CLDN6-positive advanced solid tumors. • Preliminary signals of efficacy have been observed in clinical studies; however, transient, dose-dependent elevations in inflammatory cytokines have also been reported.	NCT05262530	Stadler et al, 2024
NTLA-2001	Intellia	Transthyretin amyloidosis	CRISPR/Cas9	Phase III	• mRNA–LNP delivery of a CRISPR/Cas9-based therapy designed to inactivate the transthyretin (TTR) gene in the liver. • In the first human study, a single intravenous dose resulted in meaningful TTR knockdown, although close safety monitoring remains essential.	NCT06128629 NCT04601051	Gillmore et al, 2021
VERVE-102	Verve Therapeutics	Cardiovascular disease	CRISPR/Cas9	Phase I	• mRNA–LNP delivery of a CRISPR/Cas9-based therapy designed to inactivate the PCSK9 gene in the liver to reduce cholesterol levels. • Early clinical data demonstrated durable reductions in target protein levels in some participants; however, safety concerns led to enrollment holds and adjustments to the development program.	NCT06164730	Flight et al, 2025

### mRNA delivery of host antiviral effectors and cytokines

Innate immunity, in particular the interferon (IFN) pathway, serves as the first line of antiviral defense (Wang et al, 2017). Activation of interferon signaling triggers the transcription of interferon-stimulated genes (ISGs), which function as antiviral effectors (Xu et al, 2017). However, excessive interferon signaling can degrade exogenous mRNA and impair translation efficiency (Zhong et al, 2021; Zhong et al, 2019). As an alternative, therapeutic mRNAs can transiently express key innate immune mediators, such as type I and III interferon cytokines or ISGs, to elicit broad antiviral protection without over-activating the full pathway. Recent studies showed that mRNA–LNP encoding IFN-λ1 (namely, ETH47) achieved strong protein expression both in vitro and in vivo. Intranasal administration of ETH47 mRNA–LNP significantly protected mice against SARS-CoV-2 infection, reducing viral loads and improving clinical outcomes (Macht et al, 2024). Similarly, mRNAs encoding intrinsic antiviral ISGs, such as myxovirus resistance protein A (MxA), have been developed to suppress viral replication (Plotnikova et al, 2025).

To further broaden antiviral coverage, a recent study showed that an mRNA–LNP formulation encoding a defined set of ten ISGs conferred resistance to Zika virus, vesicular stomatitis virus, and SARS-CoV-2 in cell models (Akalu et al, 2025). This ISG set also reduced influenza A plaque size in mice and provided prophylactic protection against lethal SARS-CoV-2 challenge in hamsters (Akalu et al, 2025).

### Programmable CRISPR-based antivirals

The CRISPR–Cas system enables precise nucleic acid editing and has been repurposed as an antiviral platform. mRNA–LNP systems are well-suited for delivering CRISPR-based antivirals, offering transient and programmable expression of Cas proteins. CRISPR–Cas9 primarily targets DNA, whereas Cas13 cleaves single-stranded RNA (Tao et al, 2023), making them suitable for targeting viral DNA and RNA, respectively. In the case of HIV, CRISPR–Cas9 has been engineered to disrupt conserved regions of proviral DNA, and delivery of Cas9 mRNA with guide RNAs via LNPs produced strong and broad anti-HIV activity (Herskovitz et al, 2021). The CRISPR–Cas13 system has been explored for direct viral RNA degradation. Preclinical studies have shown that LNP-delivered Cas13 mRNA can efficiently degrade viral RNA from H1N1 influenza virus and SARS-CoV-2, suppressing replication in human respiratory cell models (Abbott et al, 2020). This approach has also been applied to other delivery systems like PBAE-based polymer (Blanchard et al, 2021) and lipid-like nanoparticles (Jiang et al, 2017), as well as to viral pathogens, such as the hepatitis B virus in animal models (Yi et al, 2023). Collectively, these studies underscore the potential of CRISPR-based antivirals as highly adaptable and sequence-specific therapeutic modalities, with early-phase clinical trials of mRNA–LNP–delivered CRISPR/Cas systems currently underway (Flight et al, 2025; Gillmore et al, 2021) (Table 2).

## Key challenges and strategic solutions

Antiviral mRNA therapeutics offer significant potential for rapid and programmable protection against viral infections. However, their clinical success hinges on overcoming key challenges, including, most notably, expanding therapeutic modalities, achieving efficient and tissue-specific delivery, managing immunogenicity, and ensuring scalable manufacturing with global accessibility. In the following sections, we explore strategic approaches to overcoming these contemporary challenges and advancing the clinical translation of mRNA-based therapeutics for viral diseases (Table 3).

**Table 3:** Potential side effects of mRNA therapeutics and proposed optimization strategies.

Potential side effect	Description/concern	Possible optimization strategies
Innate immune activation	Unwanted activation of pattern-recognition receptors (e.g., TLRs), causing inflammation or reduced translation of mRNA	Nucleoside modification (e.g., m¹Ψ), optimized UTRs and mRNA sequences, purification to remove dsRNA contaminants
Adaptive immune responses	Anti-mRNA or anti-LNP immune responses that may limit repeat dosing	Use of less immunogenic lipids and LNP formulations, alternative delivery routes (e.g., intranasal), and dosing interval optimization
LNP-related toxicity	Local or systemic reactions (e.g., inflammation, liver enzyme elevation) associated with ionizable lipids	Next-generation biodegradable or peptide-based LNPs, tissue-targeted delivery to lower systemic exposure
Off-target biodistribution	Unintended accumulation in non-target tissues (e.g., liver)	Rational lipid design (e.g., SORT LNPs), peptide-lipid targeting, ligand-based targeting, with AI tools for engineering
Potential for exaggerated cytokine responses	Overactivation of innate immunity by mRNA or adjuvant-like effects of LNPs	Lower-reactogenic mRNA–LNP formulations, controlled dosing, safety biomarkers to guide administration

### Challenge 1: limited therapeutic modalities

As emphasized, current research and development of mRNA therapeutics for viral diseases primarily focus on encoding neutralizing antibodies, host antiviral effectors, and gene-editing enzymes. Within each of these strategies, the design options for specific antiviral entities remain limited. This is partly due to the intrinsic nature of mRNA technology, which is restricted to protein production, and partly because the full potential of mRNA therapeutics has yet to be recognized and harnessed.

To effectively leverage mRNA therapeutics in this context, it is essential to systematically understand the nature of different viral pathogens (e.g., DNA vs. RNA viruses), the course of infection (e.g., acute vs. chronic), epidemiological characteristics (e.g., endemic vs. epidemic), clinical features (e.g., mild vs. severe manifestations), and therapeutic needs (e.g., treatment of affected individuals vs. societal preparedness). Over the past several decades, knowledge of virus-host interactions has expanded rapidly and continues to grow, particularly with the advent of high-throughput techniques that generate vast datasets on how host cellular machinery orchestrates the viral life cycle (See et al, 2024).

A compelling example of applying this knowledge is the cellular receptor ACE2 in SARS-CoV-2 infection. LNP-formulated mRNA encoding soluble ACE2 has demonstrated efficacy in limiting viral infection by competitively interfering with viral entry (Guimaraes et al, 2025). Since cellular receptors for a substantial proportion of viruses are already known, and efforts to identify receptors for additional viruses continue to grow (Mittler et al, 2025), the strategy of developing soluble viral decoy receptors can be extended to a broad range of viruses. These decoys function by mimicking the host receptor and competitively binding viral attachment proteins, thereby preventing the virus from interacting with target cells. As receptor identification advances, this approach becomes increasingly generalizable and may offer a scalable platform for producing antiviral interventions. Moving forward, it is important to translate this foundational knowledge into actionable therapeutic strategies to accelerate the development of targeted, adaptable mRNA-based interventions for diverse viral threats. This involves prioritizing gene candidates based on their mechanisms of action, designing optimized mRNA constructs, and rigorously testing them in relevant viral models that reflect the complexity of real-world infections.

Antiviral drug development has traditionally focused on virus-specific approaches targeting pathogens that pose an immediate health burden. However, beyond these endemic viral diseases, society remains continuously threatened by emerging viruses with epidemic potential (Dzau et al, 2025). Consequently, classical strategies fall short of meeting the demands of true pandemic preparedness, as the causative agents often remain unknown until they emerge. Broad-spectrum antivirals (BSAs) offer a promising solution by inhibiting multiple viruses from the same or different viral families. BSAs typically act on conserved viral components or host cellular pathways shared across various viruses, increasing the likelihood of efficacy against newly surfaced pathogens (Ianevski et al, 2022). In 2024, the World Health Organization (WHO) advanced pathogen prioritization by evaluating epidemic potential across 28 viral families (Mallapaty, 2024). This viral family–based framework can be leveraged to develop mRNA therapeutics with broad-spectrum activity, enhancing preparedness for future pandemics. Additionally, the WHO has identified prototype pathogens for each family, which can serve as suitable viral models for testing mRNA drug candidates.

Severe viral diseases are often accompanied by hyperinflammation driven by immune cells. This pathological response leads to extensive cell death and tissue damage, creating a vicious cycle that exacerbates morbidity and mortality (Nourazarian et al, 2025). Current antiviral therapies primarily target the virus itself and typically fail to address the associated hyperinflammation and resulting tissue injury. A recent study demonstrated that treatment with a small-molecule cell death inhibitor reduced lung inflammation and prevented mortality following influenza A virus infection, despite not inhibiting viral replication (Gautam et al, 2024). These findings suggest that key factors capable of attenuating or preventing pathological inflammation and tissue damage could be harnessed as mRNA therapeutics. However, effective treatment of such patients would likely require combination approaches that integrate these anti-inflammatory strategies with classical or mRNA-based antiviral agents.

### Challenge 2: delivery barriers of mRNA therapeutics

Pathogenic viruses exhibit diverse tissue and cell tropisms, resulting in diseases that may be localized or manifest systemically. To address this clinical complexity, mRNA therapeutics must be capable of targeted delivery across different organ systems and cell types. Achieving such precision in delivery is critical for maximizing therapeutic efficacy while minimizing off-target effects and immune-related complications. LNP delivery technology has been instrumental in advancing mRNA medicines to clinical application. However, current delivery capabilities remain suboptimal for addressing the full spectrum of viral diseases.

Currently, screening and adjustment of lipid compositions in LNPs are commonly employed to achieve tissue-specific targeting, as lipid makeup strongly influences tissue tropism, endosomal escape, and innate immune activation. One notable example is the approach known as selective organ targeting (SORT), which incorporates specific lipids into conventional LNPs to targeted delivery to organs such as the lung, spleen, and liver, as well as to relevant cell types, including epithelial cells, endothelial cells, and immune cells (Cheng et al, 2020). Recently, integrating artificial ionizable and natural amino acids into peptide-based lipids has let to the production of LNPs with predictable, tissue-specific targeting capabilities, while maintaining efficacy and safety comparable to FDA-approved formulations. These LNPs also enable efficient co-delivery of PEmax mRNA and pegRNA for prime editing in vivo (Lin et al, 2025). Neurological manifestations are serious complications of many severe viral infections that affect the central nervous system (CNS). Effective drug delivery across the blood–brain barrier (BBB) remains a major challenge in treating these conditions. Recent advances in LNP technology, more specifically, conjugation with BBB-crossing modules and amino lipids, have demonstrated enhanced efficiency of mRNA delivery to the brain compared to FDA-approved LNPs. In mouse models, these engineered LNPs have successfully transfected neurons and astrocytes throughout the brain following intravenous injection (Wang et al, 2025). Local delivery to infection sites, particularly in respiratory tract infections, offers the potential for high therapeutic efficacy while minimizing systemic side effects (Table 3). This proof-of-concept has been demonstrated through mucosal and pulmonary administration of ETH47, an LNP-formulated IFN-λ1 mRNA, in experimental models of SARS-CoV-2 infection (Macht et al, 2024). These findings support its potential translation into clinical use for treating respiratory viral infections via intranasal spray or pulmonary delivery through nebulization.

These strategies underscore the versatility of lipid chemistry, ligand engineering, and drug formulation in achieving targeted delivery to specific tissues and cells. However, identifying safe and effective LNP compositions remains labor-intensive, and the clinical translation of these novel formulations is still uncertain. For example, increased formulation complexity, such as in SORT LNPs (Cheng et al, 2020), may pose safety concerns. Low endosomal escape efficiency (less than 10%) is another critical limitation of mRNA–LNP therapeutics. The majority of internalized mRNA becomes trapped and subsequently degraded within endosomal compartments (Liu et al, 2024). A high rate of empty LNPs (ranging 40–80%) can be generated during mRNA–LNP formulation, which could limit mRNA–LNP efficiency (Li et al, 2022). In addition, certain proteins, including vitronectin, C-reactive protein, and alpha-2-macroglobulin, have been reported to interact with LNPs to form a protein corona. Recent studies suggest that while the protein corona can enhance cellular uptake, it may reduce mRNA expression by promoting lysosomal trafficking, highlighting its potential negative impact on the therapeutic performance of LNPs (Voke et al, 2025). To address these challenges, it is essential to develop optimized LNP formulations, targeted endosomal delivery strategies, and advanced analytical tools to elucidate and enhance the mechanisms governing mRNA endosomal trafficking and escape. Notably, artificial intelligence (AI)-driven tools are accelerating high-throughput virtual screening of lipid libraries and optimizing LNP formulations to enhance targeting specificity and biocompatibility (Sela et al, 2025). Alternative delivery systems, including polymeric, lipid–polymer hybrid, peptide-based, virus-like particle, and exosome-mimetic carriers, are currently under active preclinical investigation for enhanced tissue targeting and improved penetration of delivery barriers (Zhong et al, 2018). However, none have yet demonstrated a robust clinical track record for mRNA delivery. The few registered trials, such as those involving exosome-based LDLR mRNA, remain exploratory and lack published data on clinical outcomes.

### Challenge 3: safety and immunogenicity of mRNA therapeutics

Balancing immune activation with tolerability remains a significant challenge. While vaccines benefit from innate immune stimulation, therapeutic mRNAs often require suppression of excessive pattern recognition receptor signaling. Uncontrolled activation can impair translation efficiency and increase the reactogenicity of therapeutic mRNA (Zhong et al, 2018; Zhong et al, 2021). To mitigate these effects, strategies such as nucleoside modifications (e.g., pseudouridine), optimized cap structures, and advanced purification methods have been employed (Zhong et al, 2018). However, achieving the optimal balance is context-dependent. For instance, mAb expression necessitates minimal inflammation, whereas interferon-based therapies must tightly control dosing to avoid cytokine toxicity (Kowalski et al, 2019; Zhong et al, 2018). Additional safety concerns may further complicate clinical translation. Programmable nucleases like Cas13, for example, can cause collateral cleavage of host RNAs, disrupting transcriptomes and raising safety risks (Tao et al, 2023). Moreover, anti-drug antibodies may neutralize the expressed proteins or accelerate their clearance. Repeated dosing also introduces potential risks, including immune sensitization, chronic inflammation, and cumulative cytotoxicity (Pardi et al, 2018; Zhong et al, 2018). These concerns are especially relevant for long-term treatment of chronic viral infections, although may be less critical for acute viral diseases.

To address these challenges, dose-sparing RNA platforms such as aRNA, taRNA, and circRNA offer promising advantages for achieving prolonged mRNA expression in vivo (Box 1). These platforms can reduce dosing frequency and alleviate manufacturing demands, making them attractive for scalable therapeutic applications. However, their potential to elicit stronger innate immune responses, along with replicase-associated safety concerns, must be carefully evaluated and mitigated to enable safe clinical translation (Wayne and Blakney, 2024; Zhong et al, 2018). In parallel, the development of programmable, CRISPR-based antiviral systems delivered via mRNA–LNPs presents a novel therapeutic avenue (Cheng et al, 2020; Kowalski et al, 2019; Tao et al, 2023). To realize their full potential, it is critical to refine target specificity, minimize off-target effects, and enhance delivery efficiency. Continued innovation in RNA design, lipid formulation, and computational modeling will be essential to advance these systems toward clinical viability while ensuring safety and efficacy.

### Challenge 4: manufacturing, stability, and global access

Manufacturing, stability, and equitable access remain critical challenges for the widespread adoption of mRNA-based antiviral therapeutics. Although mRNA can be produced through cell-free and scalable processes, therapeutic applications often require higher or repeated dosing compared to vaccines, which increases production costs and places greater demands on cold-chain logistics. Dose-sparing platforms such as saRNA may help reduce dosing requirements, but they also raise concerns due to heightened innate immune activation, apoptosis induction, and potential cytotoxicity (Gong et al, 2024). Moreover, the long-term safety of repeated dosing remains poorly understood, with key questions surrounding immune imprinting, tolerance development, and potential effects on host gene expression requiring systematic investigation (Zhong et al, 2018).

Stability and storage represent additional limiting factors. Most current mRNA–LNP formulations require ultra-cold storage conditions, complicating distribution in low-resource or remote settings, which are often more severely affected by viral diseases (Li et al, 2024). To address this, strategies such as thermostable formulations, especially with the application of AI tools for high-throughput design of stable and thermostable mRNA (Zhang et al, 2023; Zhang et al, 2020), modification of mRNA sequences, lyophilized mRNA–LNPs, and alternative delivery carriers are under active development. For example, Comirnaty® and Spikevax® are stored frozen and incorporate 10% (w/v) sucrose as a cryoprotectant (Ruppl et al, 2025). Recent studies have shown that freeze-dried mRNA–LNPs containing 9% (w/v) trehalose and 1% (w/v) Kollidon® 12 PF (PVP) exhibit long-term stability at 2–8 °C or higher, potentially eliminating the need for ultra-cold storage (Ruppl et al, 2025).

Despite these advances, persistent challenges remain. These include ensuring batch-to-batch consistency, minimizing impurities such as double-stranded RNA, and developing robust assays to quantify therapeutic efficacy, particularly antiviral protein activity, in both preclinical and clinical settings. Ultimately, technical innovations must be aligned with global health priorities. Achieving broad impact will require integrating thermostable formulations, decentralized manufacturing capabilities, and policy frameworks that promote equitable access across diverse populations and geographies. Ensuring equitable access of mRNA therapeutics is utmost important when enhancing pandemic preparedness (Box 2), as emerging viruses are continuously emerging in resource-limited countries and settings (Treskova et al, 2025).

Box 2 Strategies to ensure affordability, accessibility, and equitability of mRNA therapeutics

- Foster strategic academia-industry collaboration: Promote partnerships between academic institutions and biotech/pharmaceutical companies to facilitate knowledge translation, accelerate development timelines, and reduce production costs through shared infrastructure and expertise.
- Engage stakeholders from resource-limited settings: Involve healthcare professionals, researchers, and policymakers from low- and middle-income countries (LMICs) to identify region-specific viral disease burdens and therapeutic needs. Support training programs for young and mid-career scientists from these regions to enable direct knowledge transfer and sustainable capacity building.
- Mobilize funding from public and philanthropic sources: Encourage investment from government agencies, international health organizations, and charitable foundations to support R&D of mRNA therapeutics targeting neglected and emerging viral diseases. Prioritize funding models that promote open-access technologies and affordable pricing.
- Streamline regulatory pathways: Collaborate with regulatory authorities to develop harmonized and expedited approval processes for mRNA therapeutics, especially during public health emergencies. Support adaptive trial designs and real-world evidence frameworks to accelerate evaluation without compromising safety.
- Implement tiered pricing and global procurement mechanisms: Develop pricing strategies that reflect countries’ economic capacities, supported by global procurement platforms (e.g., Gavi, WHO) to ensure equitable distribution and affordability across diverse populations.
- Support open science and data sharing: Encourage open-access publication of research findings, data, and protocols to democratize innovation and reduce duplication of efforts. Promote collaborative platforms for sharing preclinical and clinical data globally.
- Promote technology transfer and local manufacturing: Facilitate licensing agreements and technology transfer initiatives that enable regional production of mRNA therapeutics. Establish manufacturing hubs in LMICs to reduce dependency on imports and improve supply chain resilience.
- Ensure transparent communication and public engagement: Build public trust through transparent communication about the benefits, risks, and limitations of mRNA therapeutics. Engage communities in decision-making processes to align interventions with local values and needs.

## Future perspectives

The clinical success of mRNA vaccines has generated unprecedented momentum for expanding the platform into antiviral therapeutics. As the field evolves, therapeutic mRNA is emerging as a highly promising and adaptable modality, which is capable of rapidly encoding virtually any protein of interest, including neutralizing antibodies, antiviral peptides, cytokines, and programmable gene-editing tools such as Cas enzymes. This versatility opens avenues not only for treating active viral infections but also for targeting immune-mediated pathogenesis. Of note, combining mRNA therapeutics with agents that stimulate innate and adaptive immunity could potentially achieve synergistic antiviral effects, enhancing both the breadth and durability of protection. Nonetheless, such approaches may also increase the risk of inflammatory or off-target immune responses, thus careful optimization and vigilant monitoring are essential to balance safety and efficacy.

Crucially, the ability to design and produce mRNA constructs within weeks offers a powerful means of responding to emerging viral threats, positioning mRNA technology as a cornerstone of pandemic preparedness and a proactive strategy against “virus X.” Advances in delivery systems, such as next-generation LNPs and tissue-specific carriers, are expected to overcome current limitations related to specificity, stability, and immunogenicity. Furthermore, integrating mRNA platforms with high-throughput screening, AI-driven target discovery, and systems biology of virus-host interactions will unlock previously inaccessible antiviral pathways, accelerating the identification of novel therapeutic targets. Equipped with AI-powered tools such as RFdiffusion and ProteinMPNN (Dauparas et al, 2022; Watson et al, 2023), researchers can now design de novo proteins to counteract viral threats, which can potentially be encoded as mRNA therapeutics. For the evaluation of mRNA therapeutics, emerging human-based models, such as organoids and organ-on-chip systems, offer physiologically relevant platforms that complement conventional in vitro and animal models (Li et al, 2023; Liu et al, 2025). These advanced systems can more accurately recapitulate viral diseases and serve as essential tools for assessing the efficacy and safety profiles of mRNA therapeutics. In addition, as antiviral cytokines and CRISPR-based therapeutics exert their greatest effect during the early stages of infection, they are most effective and safe when deployed prophylactically in high-risk individuals or promptly after diagnosis. Realizing this requires rapid, sensitive diagnostics coupled with delivery platforms that enable timely, localized administration, such as intranasal or inhaled formulations. These advances support more personalized approaches, in which an individual’s exposure risk, viral load, immune profile, and circulating variant information guide both the timing and choice of antiviral intervention.

Although clinical evaluation of mRNA therapeutics remains in its early stages, ongoing preclinical and early-phase trials are laying the foundation for robust translational pipelines. Overall, the adaptability, scalability, and expanding capabilities of mRNA therapeutics signal a paradigm shift, from reactive treatment to proactive intervention, transforming how we prepare for and respond to infectious disease threats. Lastly, realizing the full potential of mRNA therapeutics in combating viral diseases will require strategic, multi-stakeholder collaboration. This includes coordinated efforts from academia, industry, funding agencies, regulatory authorities, nonprofit organizations, and the general public. Equally important is the commitment to ensuring equity throughout all stages of research, development, and implementation (Box 2).
